# Neo-Malthusianism and Eugenics*Read before the San Antonio Scientific Society, January 12, 1915.

**Published:** 1915-03

**Authors:** Malone Duggan


### Neo-Malthusianism and Eugenics.*

BY MALONE DUGGAN, M. D.

### THE CLAIMS STATED.

The old Malthusian doctrine of Malthus and Mills was one of economics. It held that the productive food supply of the country was totally inadequate to care for the natural increase of population.

Neo-Malthusianism, while accepting this principle of Malthus, differs from it materially in its application. It has for its purpose the abolition of poverty and thereby the betterment of the human race. It would substitute rational selection for natural selection. Natural selection, it claims, places the burden on the women and children, and is responsible for the multitude of ill-chosen and starving people in the world. Rational selection, on the other hand, would insure a better and more efficient stock. It

*Read before the San Antonio Scientific Society, January 12, 1915.

would, therefore, substitute for the brutal method of extermination (war and pestilence of Malthus), the more humane practice of prevention. The method employed is the conscious limitation of procreation by teaching married people the use of harmless contraceptives, especially to the poor. Marriage is encouraged by not regarding sex-union and procreation as morally inseparable. Parenthood is denied to the positively unfit and venereal infection is greatly diminished by stimulating early marriages. A fall in the birth rate follows, and consequently many of the privations of the poor are eliminated. Disease among them is lessened and a higher degree of immunity developed. It will be seen from this how strikingly similar the two propagandas are. Both would prevent propagation of the unfit. Eugenics by segregation and sterilization, Neo-Malthusianism by family limitation, resorting to sterilization only in cases where the intelligence is too low to abstain from voluntary parenthood. The only contention between them is with the propagation of the fit. The eugenists would encourage the fit to multiply. In fact, so far as possible, they would breed the human race by artificial selection the same as is done in stock and the vegetable kingdom. The Neo-Malthusians regard this as being brutal and immoral; they hold that the law of voluntary limitation should apply to all classes alike; that the number of children any family should have must be limited by their means to adequately feed, clothe and educate.

The Neo-Malthusians place the stress on quality and not quanity, and show how it is the survival rates that count rather than the birth rates. Instead of breeding, from select stock, as the eugenists would do, they "recognize that love is the biological indication of fitness for mating, and the free, sexual selection permitted by better economic conditions should produce a much better and more uniform race than can be obtained by breeding with a set purpose."

### LIMITATIONS OF EUGENICS.

Were it possible to breed characters of patriotism, courage, industry and morality so that men would be courageous, industrious and moral because of their innate qualities to be so, the plan of eugenics would be most admirable. But we are forced to observe certain limitations which preclude the carrying out of such a program. Not that we belittle the importance of eugenics in any sense, for it has its special field and is doing a most beneficent work. We are simply confronted with facts and are earnestly striving to find the method that will insure the most good.

In the first place, society must reckon with the great "mores"—the customs and prejudices which have built up character from generation to generation. This cultural phase of society is fundamental and cannot be ignored. It can only be changed through the process of evolution.

There is again the ethical point of view. It is not only the survival of the fittest, but the survival of the ethically best that must be considered in human breeding. This principle is fundamental also and precludes the possibility of human breeding ever being placed on the same footing with animal breeding.

Then there is the question of desirability. If it were possible for the race to electively breed the characters desired, there would, no doubt, be a rush for all to attain presidential qualifications and consequently the most of the race would fit very poorly in its environments.

But it is in human inheritance where lies the greatest difficulty. Though each year brings added conformation of the Mendelian principles in heredity, it is a long distance in the future before any appreciable results can come from this source. The following explanation will sufficiently indicate the trouble.

Every phase of mental and physical development is represented by a unit of quality. Certain of these qualities are heritable and others are not. The character of the person so formed depends upon the combination and interaction of these qualities upon each other. In order, then, that we may determine a desired quality we must know the unit characteristics which both parents possess, as well as those which they both lack, and the unit characters in which they differ. We must know also of each character whether it is due to a presence or absence. At present very few of the myriad of such unit characters are known; so far only general resemblances are observable. Psychical and mental units are presumably inherited the same as physical characters, but as yet this fact has not been demonstrated, and by some authorities it is claimed that psychic qualities are too complex ever to be successfully and completely resolved into their heritable units.

Havelock Ellis, speaking on race regeneration and law, remarks that: "If even the problem of the extirpation of the feeble-minded classes can be approached and largely settled on a voluntary basis, without any risky experiments in legislation, much more is this the case with the higher breeding of the race, as it may be exercised by the fully sane and responsible classes. Here is emphatically the field of the moralist, who need not feel called on to forfeit his claim to being called a moralist by clamoring for the brute force of law. Even if scientific opinion and general public opinion were ready for marriage legislation in the interests of the regeneration of the race, it would still be a problem how far such legislation is likely to be in accordance with sound morals. For legislation can only demand actions that are both generalized and externalized, and the demands of the regeneration of the race must be both particularized and internalized, or they are meaningless and even void. The law may, for instance, enact prohibitions against certain kinds of people marrying, but it cannot so prevent procreation, and the mere prohibition to marry is both unjust and unnecessary in so far as it prevents the unions of people who may be fully aware of their racial disabilities and consequent responsibilities and ready to act accordingly. Thus it is that morals is called upon to retain jealously within its own sphere these aspects of racial regeneration, and to resent the encroachments of law."

NEO-MALTHUSIANISM SUGGESTED.

In view of the limitation encountered in appealing to the eugenists for the speedy solution of the problems of race betterment we may do well to consider the claims of the Neo-Malthusians. And since no substantial gains can be expected except through recognition of the *moral* force in nature, we had as well begin to apply ourselves to those conditions that best assure this recognition. In the program proposed by the Neo-Malthusians there is little to criticize and it offers the best opportunity to achieve real race betterment.

Further strength is added to the argument in the light of the more modern conception of sex-force. The thing that distinguishes man most from the animal creation is the psychical nature of his procreative acts. Procreation with the animal kingdom is by natural selection and is controlled by an automatic physiological function. With man, procreation is not the sole function of sex. It consciously or unconsciously enters into every phase of his life and dominates his deepest interests and desires. It either builds its mansion in nobler form or brings destruction to its tenement. Therefore, if so used, it would follow that the same right that would grant unlimited exercise of this function would also give the right to regulate and control the size of the family.

Indeed, so obvious are the results to follow that one may wonder why there should be any opposition. It can only come from the old mores that has for so long decreed that sex-function was only intended for multiplying the race. No one who has had the opportunity to observe the misery and degradation that is caused from unwelcomed motherhood will remain long a believer in this orthodox teaching.

The charts here presented, prepared by Dr. C. V. Drysdale, London, show conclusively the correctness of the claims that a lower death rate follows a lower birth rate and banishes the fear that race suicide would follow family limitation. The great physical and mental improvement seen in the character of the Netherlands for the past forty years, where conscious limitation has been practiced as a national regulation, offers further evidence of its practical workings.

In the light of modern physio-chemical achievements and the promises of what might be; when the elements hydrogen and oxygen can be wrested from the air, and the bowels of earth be made to give up its carbon, there is little fear of food production not keeping pace with population. If it is contended that improved sanitation is the cause of lowering the death rate, this may be met with the claim that family limitation is the highest form of modern sanitation, and through its practice the greatest results can be accomplished. Already the better classes are voluntarily limiting their families and the greatest need now is in extending the benefits of the practice to the poorer classes.

In this day of radical changes one may well wonder whether mind is supreme, and that the will of man, after all, is the unknowable reality. When man conquers the laws of nature and converts them to his own purposes, he at least invites new and greater responsibilities, the result of which will be determined by his freedom to act and soundness of judgment.

---

Dr. Charles A. R. Campbell, whose discovery that the ordinary bat is probably the greatest destroyer of mosquitoes and other noxious insect life, has made a formal proposition to the county commissioners of San Antonio that one of his "batteries" be placed on top of the new county hospital in lieu of the regulation steeple.

---

*The American Practitioner*, New York, has been purchased by the Urologic Publishing Association and consolidated with *The American Journal of Urology, Venereal and Sexual Diseases.* The consolidated journal will be under the editorship of Dr. William J. Robinson. The publication offices will be at 12 Mt. Morris Park West, New York City.

